# Synthesis, Characterization, and In Vitro Photodynamic Activity of Novel Amphiphilic Zinc(II) Phthalocyanines Bearing Oxyethylene-Rich Substituents

**DOI:** 10.1155/2008/284691

**Published:** 2007-12-02

**Authors:** Jian-Yong Liu, Xiong-Jie Jiang, Wing-Ping Fong, Dennis K. P. Ng

**Affiliations:** ^1^Department of Chemistry and Center of Novel Functional Molecules, The Chinese University of Hong Kong, Shatin, NT, Hong Kong; ^2^Department of Biochemistry and Center of Novel Functional Molecules, The Chinese University of Hong Kong, Shatin, NT, Hong Kong

## Abstract

Three novel zinc(II) phthalocyanines substituted with one or two 3,4,5-tris(3,6,9-trioxadecoxy)benzoxy group(s) have been prepared and spectroscopically characterized. These compounds are highly soluble and remain nonaggregated in N,N-dimethylformamide. Upon excitation, they exhibit a relatively weak fluorescence emission and high efficiency to generate singlet oxygen compared with the unsubstituted zinc(II) phthalocyanine. These amphiphilic photosensitizers formulated with Cremophor EL are highly photocytotoxic against HT29 human colon adenocarcinoma and HepG2 human hepatocarcinoma cells. The mono-α-substituted analogue **4** is particularly potent with ${\text{IC}}_{50}$ values as low as 0.02 μM. The higher photodynamic activity of this compound can be attributed to its lower aggregation tendency in the culture media as shown by absorption spectroscopy and higher cellular uptake as suggested by the stronger intracellular fluorescence, resulting in a higher efficiency to generate reactive oxygen species inside the cells.

## 1. INTRODUCTION

Photodynamic therapy (PDT) has emerged as a promising modality for the treatment of malignant tumors and wet age-related macular degeneration [1–3]. It is a binary therapy that involves the combination of visible light and a photosensitizer. Each component is harmless by itself, but in combination with molecular oxygen, they result in the generation of reactive oxygen species (ROS) causing oxidative cellular and tissue damage. This treatment has several potential advantages including its minimally invasive nature, tolerance of repeated doses, and high specificity that can be achieved through precise application of the light with modern fiber-optic systems and various types of endoscopy [3]. Currently, only a few porphyrin derivatives including porfimer sodium, temoporfin, and verteporfin are clinically approved for systemic administration. These compounds, though giving a positive response in a high percentage of patients, still have various deficiencies that demand a further development of better candidates [4]. Owing to the desirable electronic absorption and photophysical properties, phthalocyanines are one of the most promising classes of candidates for this application [5]. Over the last few years, we have been interested in rational modification of this class of functional dyes with the goal of enhancing their PDT efficiency. Several new series of silicon(IV) and zinc(II) phthalocyanines have been synthesized and evaluated for their photo-physical and biological properties; see [6] and the references. As the amphiphilicity of photosensitizers is believed to have a beneficial effect on their photodynamic activity [7], amphiphilic phthalocyanines have been our targets. In this paper, we report the synthesis, photophysical properties, and in vitro photodynamic activity of three novel zinc(II) phthalocyanines bearing one or two 3,4,5-tris(3,6,9-trioxadecoxy)benzoxy substituent(s). Having three or six triethylene glycol chains on one side of the macrocycle, these compounds are amphiphilic in nature, exhibiting a high in vitro photocytotoxicity.

## 2. MATERIALS AND METHODS

### 2.1. General

All the reactions were performed under an atmosphere of nitrogen. Tetrahydrofuran (THF), n-pentanol, dichloromethane, and N,N-dimethylformamide (DMF) were distilled from sodium benzophenone ketyl, sodium, calcium hydride, and barium oxide, respectively. Chromatographic purifications were performed on silica gel (Macherey-Nagel, 70–230 mesh) columns with the indicated eluents. Size exclusion chromatography was carried out on Bio-Rad Bio-Beads S-X1 beads (200–400 mesh). All other solvents and reagents were of reagent grade and used as received. Compounds **1** and **6** were prepared as described [8].

^1^H and ^13^C{ ^1^H} NMR spectra were recorded on a Bruker DPX 300 spectrometer (^1^H, 300; ^13^C, 75.4 MHz) in CDCl_3_ or DMSO-d_6_. Spectra were referenced internally using the residual solvent [^1^H: CDCl_3_ (δ 7.26); DMSO-d_6_ (δ 2.50)] or solvent [^13^C: CDCl_3_ (δ 77.0); DMSO-d_6_ (δ 39.7)] resonances relative to SiMe_4_. Electrospray ionization (ESI) mass spectra were measured on a Thermo Finnigan MAT 95 XL mass spectrometer.

UV-Vis and steady-state fluorescence spectra were taken on a Cary 5G UV-Vis-NIR spectrophotometer and a Hitachi F-4500 spectrofluorometer, respectively. Fluorescence quantum yields $\left(\Phi_{\text{F}}\right)$ were determined by the equation: $\Phi_{\text{F}\left(\text{sample}\right)}=\left(F_{\text{sample}}/F_{\text{ref}}\right)(A_{\text{ref}}/A_{\text{sample}})\left({n_{\text{sample}}}^{2}/{n_{\text{ref}}}^{2}\right)\Phi_{\text{F}\left(\text{ref}\right)}$ [9], where F,A, and n are the measured fluorescence (area under the emission peak), the absorbance at the excitation position (610 nm), and the refractive index of the solvent, respectively. The unsubstituted zinc(II) phthalocyanine (ZnPc) in DMF was used as the reference $\left[\Phi_{\text{F}\left(\text{ref}\right)}=0.28\right]$ [10]. To minimize reabsorption of radiation by the ground-state species, the emission spectra were obtained in very dilute solutions where the absorbance at 610 nm was less than 0.03. Singlet oxygen quantum yields $\left(\Phi_{\Delta }\right)$ were measured in DMF by the method of chemical quenching of 1,3-diphenylisobenzofuran (DPBF) using ZnPc as reference $\left(\Phi_{\Delta }=0.56\right)$ [11].

### 2.2. Syntheses

#### 2.2.1. Preparation of 3-[3,4,5-tris(3,6,9-trioxadecoxy)benzoxy]phthalonitrile (2)

To a mixture of 3-nitrophthalonitrile (1.73 g, 10 mmol) and compound **1** (2.97 g, 5 mmol) in DMF (30 mL) was added anhydrous K_2_CO_3_ (6.90 g, 50 mmol). The resulting mixture was stirred at $80^{{}^{\circ}}\text{C}$ for 4 days. The solvent was then evaporated under reduced pressure and the residue was mixed with CHCl_3_ (60 mL) and water (60 mL). The aqueous layer was separated and extracted with CHCl_3_ (60 mL ×3). The combined organic layers was dried over anhydrous MgSO_4_, then evaporated to dryness. The residue was purified by silica-gel column chromatography using CHCl_3_/MeOH (60 : 1 v/v) as eluent to give compound **2** as a colorless liquid (1.41 g, 39%). ^1^H NMR: δ 7.61 (vt, J = 8.7 Hz, 1 H, ArH), 7.37 (d, J = 7.5 Hz, 1 H, ArH), 7.25 (d, J = 7.5 Hz, 1 H, ArH), 6.66 (s, 2 H, ArH), 5.17 (s, 2 H, ArCH_2_), 4.13–4.18 (m, 6 H, CH_2_), 3.85 (t, J = 4.8 Hz, 4 H, CH_2_), 3.79 (t, J = 5.1 Hz, 2 H, CH_2_), 3.70–3.74 (m, 6 H, CH_2_), 3.62–3.68 (m, 12 H, CH_2_), 3.53–3.57 (m, 6 H, CH_2_), 3.37 (two partially overlapping s, 9 H, CH_3_); ^13^C{ ^1^H} NMR (DMSO-d_6_): δ 160.9, 152.4, 137.7, 136.0, 130.7, 126.2, 119.3, 116.0, 115.6, 113.9, 107.1, 103.6, 72.0, 71.5, 71.2, 70.2, 70.0, 69.9, 69.8, 69.1, 68.5, 58.2 (some of the CH_2_ signals are overlapped); MS (ESI): an isotopic cluster peaking at m/z 743 100%, [M + Na]^+^; HRMS (ESI): m/z calcd for C_36_H_52_N_2_NaO_13_[M + Na]^+^: 743.3362, found 743.3365.

#### 2.2.2. Preparation of 4-[3,4,5-tris(3,6,9-trioxadecoxy)benzoxy]phthalonitrile (3)

According to the above procedure using 4- instead of 3-nitrophthalonitrile as a starting material, compound **3** was obtained as a colorless liquid (1.71 g, 47%). ^1^H NMR (CDCl_3_): δ 7.70 (d, J = 8.7 Hz, 1 H, ArH), 7.31 (d, J = 2.4 Hz, 1 H, ArH), 7.22 (dd, J = 2.4, 8.7 Hz, 1 H, ArH), 6.60 (s, 2 H, ArH), 5.02 (s, 2 H, ArCH_2_), 4.11–4.15 (m, 6 H, CH_2_), 3.82 (t, J = 4.8 Hz, 4 H, CH_2_), 3.76 (t, J = 5.1 Hz, 2 H, CH_2_), 3.68–3.71 (m, 6 H, CH_2_), 3.60–3.65 (m, 12 H, CH_2_), 3.50–3.53 (m, 6 H, CH_2_), 3.34 (s, 9 H, CH_3_); ^13^C{ ^1^H} NMR (CDCl_3_): δ 161.5, 152.9, 138.7, 135.2, 129.7, 120.0, 119.6, 117.3, 115.5, 115.1, 107.5, 107.2, 72.2, 71.8, 71.0, 70.7, 70.6, 70.4, 69.6, 68.9, 58.9 (some of the CH_2_ signals are overlapped); MS (ESI): an isotopic cluster peaking at m/z 743 100%, [M + Na]^+^; HRMS (ESI): m/z calcd for C_36_H_52_N_2_NaO_13_[M + Na]^+^: 743.3362, found 743.3361.

#### 2.2.3. Preparation of phthalocyanine (4)

A mixture of phthalonitrile **2** (0.26 g, 0.36 mmol), unsubstituted phthalonitrile (0.46 g, 3.59 mmol), and Zn(OAc)_2_ *·* 2H_2_O (0.22 g, 1.00 mmol) in n-pentanol (15 mL) was heated to $100^{{}^{\circ}}\text{C}$, then a small amount of 1,8-diazabicyclo[5.4.0]undec-7-ene (DBU) (0.5 mL) was added. The mixture was stirred at 140–$150^{{}^{\circ}}\text{C}$ for 24 hours After a brief cooling, the volatiles were removed under reduced pressure. The residue was dissolved in CHCl_3_ (120 mL), then filtered to remove part of the unsubstituted zinc(II) phthalocyanine formed. The filtrate was collected and evaporated to dryness in vacuo. The residue was purified by silica-gel column chromatography using CHCl_3_/CH_3_OH (30 : 1 v/v) as eluent, followed by size exclusion chromatography using THF as eluent. The crude product was further purified by recrystallization from a mixture of THF and hexane (0.11 g, 26%). ^1^H NMR (CDCl_3_ with a trace amount of pyridine-d_5_): δ 9.41–9.46 (m, 5 H, Pc-${\text{H}}_{\alpha }$), 9.16 (d, J = 7.5 Hz, 1 H, Pc-${\text{H}}_{\alpha }$), 9.13 (d, J = 6.9 Hz, 1 H, Pc-${\text{H}}_{\alpha }$), 8.07–8.15 (m, 7 H, Pc-H_*β*_), 7.69 (d, J = 7.8 Hz, 1 H, Pc-H_*β*_), 7.30 (s, 2 H, ArH), 5.80 (s, 2 H, ArCH_2_), 4.31 (t, J = 5.1 Hz, 2 H, CH_2_), 4.19 (t, J = 5.1 Hz, 4 H, CH_2_), 3.90 (t, J = 5.1 Hz, 2 H, CH_2_), 3.78–3.82 (m, 2H, CH_2_), 3.66–3.75 (m, 8 H, CH_2_), 3.55–3.59 (m, 6 H, CH_2_), 3.47–3.51 (m, 8 H, CH_2_), 3.38–3.41 (m, 7 H, CH_2_ and CH_3_), 3.24 (s, 6 H, CH_3_); ^13^C{ ^1^H} NMR (DMSO-d_6_): δ 155.6, 152.7, 152.6, 152.3, 152.2, 140.3, 138.4, 137.9, 137.7, 137.6, 133.5, 130.6, 129.1, 125.3, 122.5, 122.4, 122.1, 115.3, 114.0, 107.8, 72.3, 71.6, 71.5, 71.0, 70.2, 70.0, 69.9, 69.8, 69.3, 68.8, 58.3, 58.2 (some of the signals are overlapped); MS (ESI): an isotopic cluster peaking at m/z 1191 100%, [M + Na]^+^; HRMS (ESI): m/z calcd for C_60_H_64_N_8_NaO_13_Zn [M + Na]^+^: 1191.3777, found 1191.3783.

#### 2.2.4. Preparation of phthalocyanine (5)

According to the above procedure, phthalonitrile **3** (0.26 g, 0.36 mmol) was treated with unsubstituted phthalonitrile (0.46 g, 3.59 mmol) and Zn(OAc)_2_ *·*2H_2_O (0.22 g, 1.00 mmol) to give phthalocyanine **5** as a blue solid (0.09 g, 21%). ^1^H NMR (CDCl_3_ with a trace amount of pyridine-d_5_): δ 9.20–9.35 (m, 6 H, Pc-H_*α*_), 9.05 (d, J = 7.8 Hz, 1 H, Pc-H_*α*_), 8.66 (s, 1 H, Pc-H_*α*_), 8.06–8.12 (m, 6 H, Pc-H_*β*_), 7.62 (d, J = 8.4 Hz, 1 H, Pc-H_*β*_), 6.99 (s, 2 H, ArH), 5.46 (s, 2 H, ArCH_2_), 4.33 (t, J = 4.8 Hz, 4 H, CH_2_), 4.24 (t, J = 5.1 Hz, 2 H, CH_2_), 3.94 (t, J = 4.8 Hz, 4 H, CH_2_), 3.86 (t, J = 4.8 Hz, 2 H, CH_2_), 3.76–3.80 (m, 6 H, CH_2_), 3.62–3.71 (m, 12 H, CH_2_), 3.53–3.57 (m, 6 H, CH_2_), 3.38 (s, 3 H, CH_3_), 3.36 (s, 6 H, CH_3_); ^13^C{ ^1^H} NMR (DMSO-d_6_): δ 159.9, 152.6, 152.4, 152.3, 152.2, 152.0, 151.7, 151.6, 139.5, 137.8, 137.5, 132.7, 130.7, 129.0, 128.9, 123.0, 122.2, 117.8, 107.2, 105.6, 72.1, 71.5, 70.3, 70.1, 70.0, 69.9, 69.4, 68.8, 58.3, 55.1 (some of the signals are overlapped); MS (ESI): an isotopic cluster peaking at m/z 1191 95%, [M + Na]^+^; HRMS (ESI): m/z calcd for C_60_H_64_N_8_NaO_13_Zn [M + Na]^+^: 1191.3777, found 1191.3771.

#### 2.2.5. Preparation of 3,6-bis[3,4,5-tris(3,6,9-trioxadecoxy)benzoxy]phthalonitrile (7)

A mixture of compound **6** (2.06 g, 3.36 mmol), 2,3-dicyanohydroquinone (0.27 g, 1.69 mmol), and K_2_CO_3_ (1.17 g, 8.47 mmol) in DMF (10 mL) was stirred at $100^{{}^{\circ}}\text{C}$ for 24 hours The volatiles were then removed under reduced pressure. The residue was mixed with water (50 mL) and the mixture was extracted with CHCl_3_ (50 mL × 3). The combined organic extracts was dried over anhydrous MgSO_4_ and evaporated under reduced pressure. The residue was then purified by silica-gel column chromatography using CHCl_3_/MeOH (20:1 v/v) as eluent to give the product as a pale yellow transparent liquid (1.95 g, 88%). ^1^H NMR (CDCl_3_): δ 7.13 (s, 2 H, ArH), 6.64 (s, 4 H, ArH), 5.07 (s, 4 H, ArCH_2_), 4.12–4.17 (m, 12 H, CH_2_), 3.84 (t, J = 4.8 Hz, 8 H, CH_2_), 3.78 (t, J = 5.1 Hz, 4 H, CH_2_), 3.71–3.74 (m, 12 H, CH_2_), 3.62–3.67 (m, 24 H, CH_2_), 3.52–3.57 (m, 12 H, CH_2_), 3.37 (two partially overlapping s, 18 H, CH_3_); ^13^C{ ^1^H} NMR (CDCl_3_): δ 154.7, 152.8, 138.2, 130.3, 119.4, 112.9, 106.4, 105.7, 72.2, 71.7, 71.6, 70.6, 70.5, 70.3, 69.5, 68.7, 58.8 (some of the CH_2_ signals are overlapped); MS (ESI): an isotopic cluster peaking at m/z 1336 100%, [M + Na]^+^; HRMS (ESI): m/z calcd for C_64_H_100_N_2_NaO_26_ [M + Na]^+^: 1335.6457, found 1335.6462.

#### 2.2.6. Preparation of phthalocyanine (8)

According to the procedure described for **4**, phthalonitrile **7** (0.50 g, 0.38 mmol) was treated with unsubstituted phthalonitrile (0.49 g, 3.82 mmol) and Zn(OAc)_2_ *·*2H_2_O (0.23 g, 1.05 mmol) to give phthalocyanine **8** as a blue-green oil (54 mg, 8%). ^1^H NMR (CDCl_3_): δ 9.43–9.47 (m, 4 H, Pc-H_*α*_), 9.20 (d, J = 7.5 Hz, 2 H, Pc-H_*α*_), 8.03–8.15 (m, 6 H, Pc-H_*β*_), 7.68 (s, 2 H, Pc-H_*β*_), 7.37 (s, 4 H, ArH), 5.90 (s, 4 H, ArCH_2_), 4.05 (t, J = 5.1 Hz, 4 H, CH_2_), 3.80 (t, J = 5.1 Hz, 8 H, CH_2_), 3.70 (t, J = 5.1 Hz, 4 H, CH_2_), 3.61–3.65 (m, 4 H, CH_2_), 3.55–3.58 (m, 8 H, CH_2_), 3.44–3.48 (m, 12 H, CH_2_), 3.34–3.37 (m, 12 H, CH_2_), 3.30 (s, 6 H, CH_3_), 3.18–3.21 (m, 8 H, CH_2_), 3.12 (s, 12 H, CH_2_), 2.90 (s, 12 H, CH_3_); ^13^C{ ^1^H} NMR (CDCl_3_): 153.6, 153.4, 153.3, 152.5, 152.2, 150.2, 138.6, 138.3, 138.1, 137.5, 133.6, 129.0, 128.8, 128.6, 128.5, 122.6, 122.3, 122.1, 116.5, 107.0, 72.3, 72.2, 71.8, 71.3, 70.5, 70.4, 69.9, 69.7, 69.2, 68.1, 58.8, 58.4 (some of the CH_2_ signals are overlapped); MS (ESI): an isotopic cluster peaking at m/z 1784 20%, [M + Na]^+^; HRMS (ESI): m/z calcd for C_88_H_112_N_8_NaO_26_Zn [M + Na]^+^: 1783.6871, found 1783.6862.

### 2.3. In vitro studies

#### 2.3.1. Cell lines and culture conditions

The HT29 human colorectal carcinoma cells (from ATCC, no. HTB-38) were maintained in Dulbecco's modified Eagle's medium (DMEM) (Invitrogen, cat no. 10313-021) supplemented with fetal calf serum (10%), penicillin-streptomycin (100 units mL^-1^ and 100 mgmL^-1^, resp.), L-glutamine (2 mM), and transferrin (10 mgmL^-1^). The HepG2 human hepatocarcinoma cells (from ATCC, no. HB-8065) were maintained in RPMI medium 1640 (Invitrogen, cat no. 23400-021) supplemented with fetal calf serum (10%) and penicillin-streptomycin (100 units mL^-1^ and 100 mgmL^-1^, resp.). Approximately $3\times 10^{4}$ (for HT29) or $4\times 10^{4}$ (for HepG2) cells per well in these media were inoculated in 96-multiwell plates and incubated overnight at $37^{{}^{\circ}}\text{C}$ in a humidified 5% CO_2_ atmosphere.

#### 2.3.2. Photocytotoxicity assay

Phthalocyanines **4**, **5**, and **8** were first dissolved in DMF to give 1.5 mM solutions, which were diluted to 80 μM with an aqueous solution of Cremophor EL (Sigma, 0.47 g in 100 mL of water). The solutions were filtered with a 0.2 μm filter, then diluted with the culture medium to appropriate concentrations (two-fold dilutions from 8 μM). The cells, after being rinsed with phosphate buffered saline (PBS), were incubated with 100 μL of these phthalocyanine solutions for 2 hours at $37^{{}^{\circ}}\text{C}$ under 5% CO_2_. The cells were then rinsed again with PBS and refed with 100 μL of the culture medium before being illuminated at ambient temperature. The light source consisted of a 300 W halogen lamp, a water tank for cooling, and a color glass filter (Newport) cut-on 610 nm. The fluence rate (λ>610 nm) was 40 mW cm^-2^. An illumination of 20 minutes led to a total fluence of 48 J cm^-2^.

Cell viability was determined by means of the colorimetric MTT assay [12]. After illumination, the cells were incubated at $37^{{}^{\circ}}\text{C}$ under 5% CO_2_ overnight. An MTT (Sigma) solution in PBS (3 mgmL^-1^, 50 μL) was added to each well followed by incubation for 2 hours under the same environment. A solution of sodium dodecyl sulfate (SDS, Sigma) (10% by weight, 50 μL) was then added to each well. The plate was incubated in an oven at $60^{{}^{\circ}}\text{C}$ for 30 minutes, then 80 μL of *iso*-propanol was added to each well. The plate was agitated on a Bio-Rad microplate reader at ambient temperature for 10 sec before the absorbance at 540 nm at each well was taken. The average absorbance of the blank wells, which did not contain the cells, was subtracted from the readings of the other wells. The cell viability was then determined by the equation: % Viability = $[\Sigma \left({\text{A}}_{i}/{\text{A}}_{\text{control}}\times 100)\right]/n$, where A*_i_* is the absorbance of the ith data (i=1,2,…,n), ${\text{A}}_{\text{control}}$ is the average absorbance of the control wells, in which the phthalocyanine was absent, and n (= 4) is the number of the data points.

#### 2.3.3. Fluorescence microscopic studies

For the detection of the intracellular fluorescence intensity of compounds **4**, **5**, and **8**, approximately $1.2\times 10^{5}$ HT29 cells in the culture medium (2 mL) were seeded on a coverslip (diameter = 25 mm) and incubated overnight at $37^{{}^{\circ}}\text{C}$ under 5% CO_2_. The medium was removed, then the cells were incubated with 2 mL of an 8 μM phthalocyanine dilution in the medium for 2 h under the same conditions. The cells were then rinsed with PBS and viewed with an Olympus IX 70 inverted microscope. The excitation light source (at 630 nm) was provided by a multiwavelength illuminator (Polychrome IV, TILL Photonics). The emitted fluorescence (*>*660 nm) was collected using a digital cooled CCD camera (Quantix, Photometrics). Images were digitalized and analyzed using MetaFluor V.4.6 (Universal Imaging).

## 3. RESULTS AND DISCUSSION

### 3.1. Molecular design and chemical synthesis

Zinc(II) phthalocyanines are good candidates for PDT application. In addition to their relatively high stability, the closed-shell zinc(II) center imparts desirable photophysical characteristics to the macrocycles [13]. Introduction of substituents at the peripheral positions can also tailor the properties of the macrocycles such as their solubility in biological media, aggregation behavior, and targeting properties. As a result, zinc(II) phthalocyanines have received considerable attention as efficient photosensitizers [5]. We describe herein three novel zinc(II) phthalocyanines (compounds **4**, **5**, and **8**) which contain the bulky and hydrophilic 3,4,5-tris(3,6,9-trioxadecoxy)benzoxy moiety. Having one or two of these substituents, the *π*-*π* stacking tendency is reduced and the macrocycles become amphiphilic in nature. These properties should be advantageous for singlet oxygen generation and cellular uptake, by which the photodynamic activity can be enhanced. The relatively rare 1,4-disubstituted phthalocyanine **8** also has a longer Q-band maximum compared with the *α*- and *β*-monosubstituted counterparts, which is also an advantage that can increase the light penetration depth [14].

**Scheme 1:**
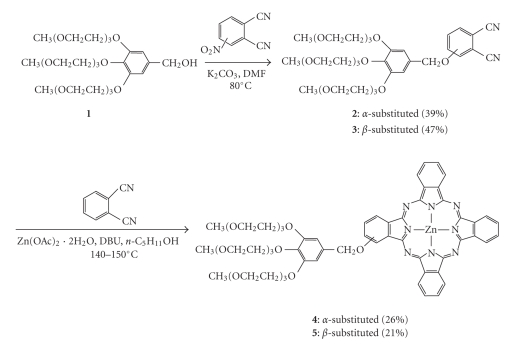


**Scheme 2:**
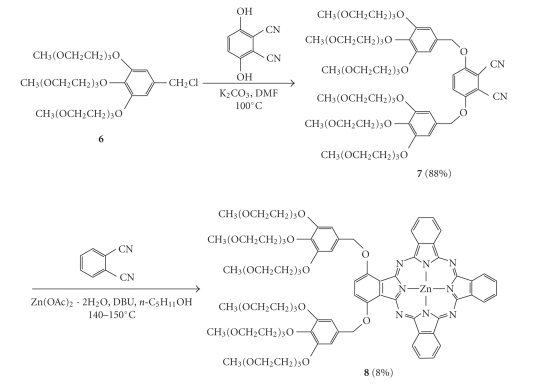


Scheme 1 shows the synthetic route used to prepare the monosubstituted phthalocyanines **4** and **5**. Reaction of benzyl alcohol **1** with 3- or 4-nitrophthalonitrile in the presence of K_2_CO_3_ in DMF gave the substituted phthalonitrile **2** or **3**, respectively. These compounds then underwent a mixed cyclization with an excess of the unsubstituted phthalonitrile (10 equiv.) in the presence of Zn(OAc)_2_ *·*2H_2_O and DBU in n-pentanol to afford the corresponding “3+1” products **4** and **5**. These reactions also produced the unsubstituted ZnPc as a major side-product, which could be separated readily by filtration and chromatography as a result of its lower solubility and slower mobility in the silica gel column. During the chromatographic purification, a trace amount of some other blue products was also separated, but no attempt was made to characterize these minor side-products. Similarly, treatment of 2,3-dicyanohydroquinone with benzyl chloride **6** and K_2_CO_3_ afforded dinitrile **7**, which was then cyclized with the unsubstituted phthalonitrile in the presence of Zn(OAc)_2_ *·*2H_2_O to give **8** (Scheme 2). All these zinc(II) phthalocyanines were soluble in common organic solvents and possessed high stability, which facilitated the purification by silica-gel column chromatography, size exclusion chromatography, followed by recrystallization.

### 3.2. Spectroscopic characterization and photophysical properties

**Figure 1:**
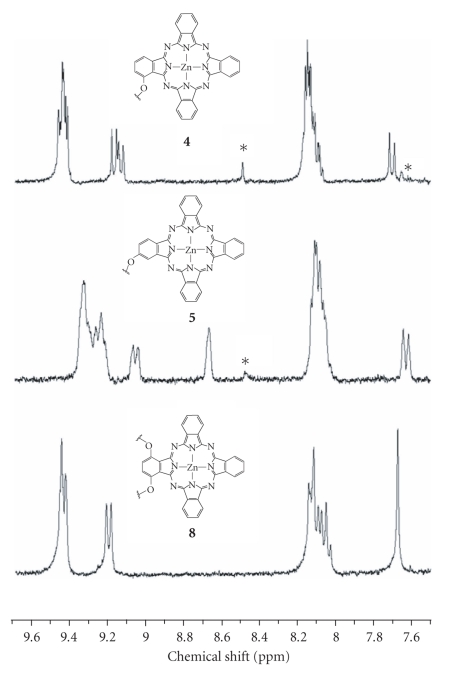
The aromatic region of the ^1^H NMR spectra of **4**, **5**, and **8** in CDCl_3_; * indicates the trace amount of pyridine-d_5_ added for the former two complexes.

All the new compounds were fully characterized with various spectroscopic methods. The NMR signals for the phthalocyanine ring protons of **4**, **5**, and **8** are very distinct in CDCl_3_ (with a trace amount of pyridine-d_5_ for the former two complexes to reduce their aggregation), which provide a useful means for characterization. As shown in Figure 1, the ^1^H NMR spectrum of the *α*-substituted phthalocyanine **4** shows a multiplet at δ 9.41–9.46 (5 H) and two doublets at δ 9.16 (1 H) and 9.13 (1 H) for the 7 phthalocyanine *α* protons. The 8 *β* protons resonate as a multiplet at δ 8.07–8.15 (7 H) and a doublet at δ 7.69 (1 H). For the *β*-analogue **5**, a multiplet at δ 9.20–9.35 (6 H), a doublet at δ 9.05 (1 H), and a singlet at δ 8.66 (1 H) are seen for the 8 phthalocyanine *α* protons, while the signals for the 7 *β* protons appear as a multiplet at δ 8.06–8.12 (6 H) and a doublet at δ 7.62 (1 H). Phthalocyanine **8** has a $C_{2\text{v}}$ symmetry. The doublet at δ 9.20 can be assigned to the two phthalocyanine *α* ring protons close to the benzoxy groups, while the multiplet at δ 9.43–9.47 is due to the remaining four *α* protons. The singlet at δ 7.68 can be readily assigned to the two *β* protons adjacent to the benzoxy groups, while the multiplet at δ 8.03–8.15 is attributed to the remaining six *β* protons.

The ^13^C NMR data of these compounds were also in accord with the structures though some of the phthalocyanine ring carbon signals (for **4** and **5**) and the chain CH_2_ signals were overlapped. For compound **8**, a total of 20 signals were observed in the aromatic region (δ 107.0–153.6) for the 16 phthalocyanine and 4 benzene ring carbons, which is consistent with the $C_{2\text{v}}$ symmetry.

The ESI mass spectra of these phthalocyanines were also recorded. The molecular ion [M + Na]^+^ isotopic cluster could be detected in all the cases. The isotopic distribution was in good agreement with the corresponding simulated pattern. The identity of these species was also confirmed by accurate mass measurements.

**Figure 2:**
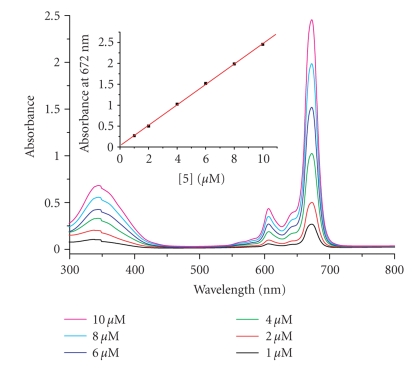
UV-Vis spectra of **5** in DMF. The inset plots the Q-band absorbance versus the concentration of **5**.

The electronic absorption and basic photophysical data of phthalocyanines **4**, **5**, and **8** were measured in DMF and are summarized in Table 1. All the three compounds gave very similar UV-Vis spectra, which are typical for nonaggregated phthalocyanines. The spectrum of compound **5**, for example, showed the B-band at 344 nm, a vibronic band at 606 nm, and an intense and sharp Q-band at 672 nm, which strictly followed the Lambert Beer's law (Figure 2). Upon excitation at 610 nm, the compound is emitted at 677 nm with a fluorescence quantum yield of 0.19. Substitution at the *α* position (compound **4**) slightly shifted the Q-band and fluorescence emission to the red by 4-5 nm. Introduction of an additional *α*-substituent (compound **8**) further shifted the Q-band to 690 nm and the fluorescence emission to 696 nm.

**Figure 3:**
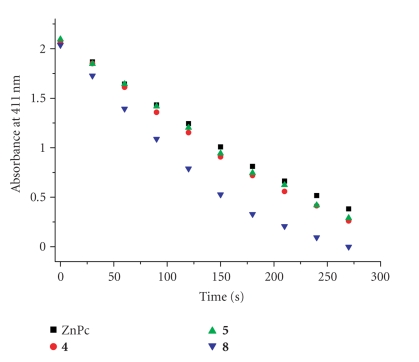
Comparison of the rates of decay of DPBF in DMF, as monitored spectroscopically at 411 nm, using phthalocyanines **4**, **5**, and **8** as the photosensitizers and ZnPc as the reference.

The singlet oxygen quantum yields ($\Phi_{\Delta }$) of these compounds were also determined using 1,3-diphenylisobe-nzofuran (DPBF) as the scavenger. The concentration of the quencher was monitored spectroscopically at 411 nm along with time, from which the values of $\Phi_{\Delta }$ could be determined by the method described previously [11]. These data are also summarized in Table 1. Figure 3 compares the rates of decay of DPBF using these compounds and ZnPc as the photosensitizers. It can be seen that all these phthalocyanines are efficient singlet oxygen generators, particularly the 1,4-disubstituted analogue **8**, of which the value of $\Phi_{\Delta }$ (0.84) is significantly higher than that of ZnPc (0.56), which was used as the reference.

### 3.3. In vitro photodynamic activity

**Figure 4:**
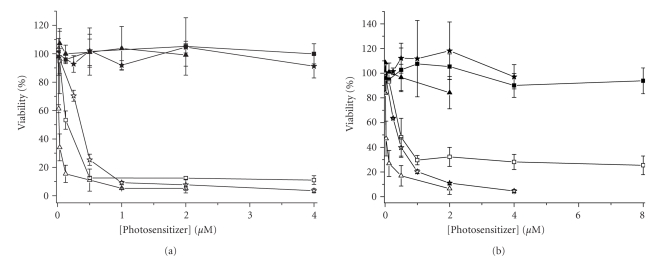
Effects of **4** (triangles), **5** (stars), and **8** (squares) on (a) HT29 and (b) HepG2 in the absence (closed symbols) and presence (open symbols) of light (*λ* *>* 610 nm, 40 mW cm^-2^, 48 J cm^-2^). Data are expressed as mean value ± SEM of three independent experiments, each performed in quadruplicate.

The in vitro photodynamic activity of photosensitizers **4**, **5**, and **8** in Cremophor EL emulsions was investigated against two different cell lines, namely, HT29 human colorectal carcinoma and HepG2 human hepatocarcinoma cells. As shown in Figure 4, the three compounds are essentially noncytotoxic in the absence of light, but exhibit a very high photocytotoxicity. The corresponding IC_50_ values are summarized in Table 2. It can be seen that all these compounds are highly potent and the effects on HT29 are greater than those on HepG2. The phthalocyanine **4** is particularly potent with the IC_50_ values down to 0.02 μM. About 1 μM of the dye is sufficient to kill 90% of the cells.

**Figure 5:**
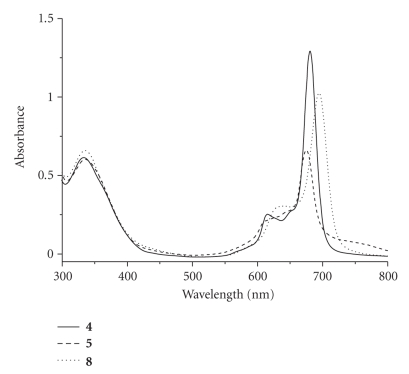
Electronic absorption spectra of **4** (solid line), **5** (dashed line), and **8** (dotted line), formulated with Cremophor EL, in the DMEM culture medium (all at 8 *μ*M).

It is worth noting that although phthalocyanine **4** exhibits a relatively lower singlet oxygen quantum yield than the other two analogues in DMF (Table 1), its photocytotoxicity is the highest among the three photosensitizers (Table 2). To account for the results, the absorption spectra of these compounds in the culture media were recorded. As shown in Figure 5, the Q-band for compound **4** in the DMEM medium (for HT29) remains very sharp and intense, while those for **5** and **8** are weaker and broadened. Very similar results were obtained in the RPMI medium (for HepG2). This is a strong indication that compound **4** is significantly less aggregated in these media, which should lead to a higher photosensitizing efficiency.

**Figure 6:**
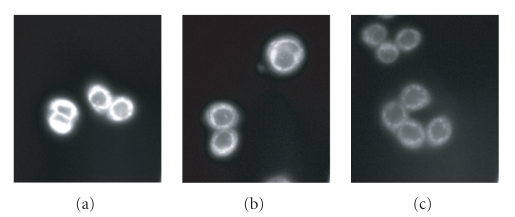
Fluorescence microscopic images of HT29 tumor cells after incubation with (a) **4**, (b) **5**, and (c) **8** at a concentration of 8 μM for 2 hours.

To further explain the photocytotoxicity results, fluorescence microscopic studies were also carried out to shed light on the cellular uptake of photosensitizers **4**, **5**, and **8**. After incubation with these compounds (formulated with Cremophor EL) for 2 hours and upon excitation at 630 nm, the HT29 cells showed intracellular fluorescence throughout the cytoplasm as shown in Figure 6, indicating that there was a substantial uptake of the dyes. The qualitative fluorescence intensity follows the order **4** *>* **5** *>* **8**, suggesting that the *α*-substituted phthalocyanine **4** also has the highest uptake among the three photosensitizers. This may also account for the highest photocytotoxicity of this compound.

In conclusion, we have prepared and characterized three novel “3+1” zinc(II) phthalocyanines substituted with one or two 3,4,5-tris(3,6,9-trioxadecoxy)benzoxy group(s). These compounds exhibit a high photocytotoxicity against HT29 and HepG2 cells with IC_50_ values down to 0.02 μM. The mono-*α*-substituted phthalocyanine **4** is more potent than the other two analogues, which can be partly explained by its lower aggregation tendency in the culture media and higher cellular uptake as shown by absorption spectroscopy and fluorescence microscopy, respectively.

## Figures and Tables

**Table 1:** Electronic absorption and photophysical data for **4**, **5**, and **8** in DMF.

Compound	$\lambda_{\max}$ (nm) (log ε)	$\lambda_{\text{em}}$ (nm)${}^{\text{(a)}}$	Φ_F_^(b)^	Φ_Δ_^(c)^
**4**	334 (4.69), 611 (4.58), 677 (5.40)	681	0.20	0.60
**5**	344 (4.79), 606 (4.62), 672 (5.39)	677	0.19	0.62
**8**	337 (4.73), 621 (4.55), 690 (5.31)	696	0.14	0.84

^(a)^Excited at 610 nm. ^(b)^Using unsubstituted zinc(II) phthalocyanine (ZnPc) in DMF as the reference $\left(\Phi_{\text{F}}=0.28\right)$. ^(c)^Using ZnPc as the reference ($\Phi_{\Delta }$ = 0.56 in DMF).

**Table 2:** Comparison of the IC_50_ values${}^{\text{(a)}}$ of phthalocyanines **4**, **5**, and **8** against HT29 and HepG2.

Compound	For HT29 (*μ*M)	For HepG2 (*μ*M)
**4**	0.02	0.03
**5**	0.36	0.39
**8**	0.15	0.49

^(a)^Defined as the dye concentration required to kill 50% of the cells.
